# Supplementation of Adult Rats with Moderate Amounts of β-Carotene Modulates the Redox Status in Plasma without Exerting Pro-Oxidant Effects in the Brain: A Safer Alternative to Food Fortification with Vitamin A?

**DOI:** 10.3390/nu6125572

**Published:** 2014-12-01

**Authors:** Carlos Eduardo Schnorr, Maurilio da Silva Morrone, André Simões-Pires, Leonardo da Silva Bittencourt, Fares Zeidán-Chuliá, José Cláudio Fonseca Moreira

**Affiliations:** Centro de Estudos em Estresse Oxidativo, Departamento de Bioquímica, Instituto de Ciências Básicas da Saúde, Universidade Federal do Rio Grande do Sul, Rua Ramiro Barcelos 2600, Anexo Depto. Bioquímica, Lab 32, CEP 90035-003, Porto Alegre, Brazil; E-Mails: maurilio.bio@gmail.com (M.S.M); andresimoespires@gmail.com (A.S.-P.); lsbittencourt@hotmail.com (L.S.B); fzchulia.biomed@gmail.com (F.Z.-C.); 00006866@ufrgs.br (J.C.F.M.)

**Keywords:** vitamin A, rat model, oxidative stress, retinol, toxicity, nutrition

## Abstract

Despite the antioxidant potential of vitamin A, recent studies reported that chronic retinol ester supplementation can also exert pro-oxidant effects and neurotoxicity *in vivo* and raises the mortality rates among healthy subjects. Our aim was to find evidence for a safer (*i.e.*, less toxic) molecule with provitamin A activity. Therefore, we investigated whether chronic supplementation of healthy Wistar rats with β-carotene (0.6, 3, and 6 mg/kg/day) would demonstrate antioxidant characteristics without leading to pro-oxidant side effects in the brain. Total reactive antioxidant potential (TRAP), thiobarbituric reactive species level (TBARS), and total reduced thiol content (SH) were evaluated in plasma. TBARS and SH were additionally evaluated in selected brain regions together with superoxide dismutase (SOD) and catalase (CAT) activity. In the present study, we show that β-carotene is able to exert antioxidant activity in plasma without triggering pro-oxidant events in the brain, providing evidence that may justify its further evaluation as a safer nutritional supplement with provitamin A activity.

## 1. Introduction

Food fortification and dietary supplementation with retinol esters are commonly used to mitigate the impacts of hypovitaminosis A. However, increasing interest in fortified foods and vitamin A supplements is leading to a large percentage of the healthy population being constantly exposed to a higher intake of vitamin A than recommended [1]. Additionally, retinol esters are widely proposed as therapeutic agents for the treatment of many diseases and disorders, such as depression, schizophrenia, and Alzheimer’s disease [2]. However, excessive consumption of vitamin A can have a severe adverse impact on human health. Even without any manifestation of clinical signs of hypervitaminosis A, neurotoxic effects may occur in adults due to excessive vitamin A intake because vitamin A readily enters the central nervous system (CNS) [3]. Recently, the harmful effects of retinol ester supplementation to the brain have been investigated using experimental animal models. Retinol ester supplementation at doses ranging from 600 to 3000 retinol activity equivalents (RAE) was demonstrated to induce pro-oxidant effects in different regions of the CNS, including the hippocampus, striatum, and cerebral cortex [4,5,6,7]. Therefore, the search for safer sources of vitamin A seems to be timely and necessary.

To the best of our knowledge, no previous studies have ever investigated the effects of supplementation with provitamin A carotenoids on the brain. In addition to its potential as an alternative source of vitamin A, β-carotene, of all the dietary provitamin A carotenoids, appears to be an excellent choice as vitamin A source because it displays the greatest provitamin A activity among all other studied carotenoids [8]. Additionally, β-carotene is proposed to be a natural antioxidant able to trap and neutralize free radicals and prevent oxidative stress [9]. Moreover, despite the acute and chronic toxic effects associated with retinol ester supplementation, β-carotene has not been associated with hypervitaminosis A even when administered at doses up to 3000 RAE [8]. Thus, the aim of the present study was (i) to investigate whether chronic oral supplementation with β-carotene (by orogastric gavage for 28 days) to well-fed male Wistar rats would demonstrate systemic antioxidant potential at the therapeutic doses of 0.6, 3 and 6 mg/kg/day; and (ii) to investigate the safety of such administration for the CNS by evaluating any potential vitamin A-associated neurotoxicity (pro-oxidant effects) of these same doses to the vulnerable regions of the brain (hippocampus, striatum, and cerebral cortex).

## 2. Experimental Section

### 2.1. Animals

Male Wistar rats (110–120 day old *Rattus novergicus*) were obtained from our breeding colony and housed in groups of four animals on a 12 h light–dark cycle (lights on at 7:00 AM) at constant temperature (22 ± 4 °C) and relative humidity (30%–70%). Standard food (CR1 lab chow, Nuvilab Ltda., Curitiba, Brazil) and water were provided *ad libitum*. The Federal University of Rio Grande do Sul Ethical Committee for animal experimentation reviewed and approved the study protocol (project number 21563). All experimental procedures were in compliance with the recommendations of the Brazilian Society for Science in Laboratory Animals (SBCAL-COBEA) and the National Institute of Health Guide for the Care and Use of Laboratory Animals [10].

### 2.2. Treatment

The animals were randomized into four study groups (*n* = 6 or 7 animals per group) and received vegetable oil (control group) or β-carotene (0.6, 3, or 6 mg/kg/day) for 28 days orally, via a metallic gastric tube (gavage). The animals were weighed once a week and received the gavage in a maximum volume of 0.3 mL. A 10 mg/mL stock solution of β-carotene (Sigma-Aldrich^®^, St. Louis, MO, USA) was prepared in vegetable oil (Cargill Inc., Minneapolis, MN, USA) and was protected from light. The retinol activity equivalent (RAE) of β-carotene in this formulation was 300, 600, and 3000 RAE/kg/day, respectively, considering the actual rate to be 2 μg of β-carotene to 1 μg retinol for synthetic pure β-carotene prepared in oil [11,12]. The treatments were administered at the beginning of the dark phase (8:00 PM) to ensure maximum β-carotene absorption because this nutrient is better absorbed during or after a meal.

### 2.3. Biochemical Analyses

All animals were killed by decapitation 24 h after the last β-carotene administration. Blood samples were collected, and plasma for analysis was separated immediately (2000× *g* per 10 min). The cerebral cortex, hippocampus, and striatum from each animal were separated by dissection on ice and homogenized in 50 mM potassium phosphate buffer (KPB) pH 7.4. The samples were centrifuged (10,000× *g*, 10 min), the supernatants were collected and all redox results were normalized to the protein content [13].

Thiobarbituric acid-reactive species (TBARS) formation was evaluated as an estimation of the oxidative damage to lipids as previously described [14]. TBARS were determined by the absorbance at 532 nm, and the results were expressed as pmol or nmol TBARS/mg protein (for plasma and dissected tissues, respectively). Total thiol content was evaluated as an estimation of the oxidative damage to proteins, as previously described [15]. Total thiol content was determined by the absorbance after 60 min at 412 nm, and the results were expressed as μmol SH/mg protein. Plasma total reactive antioxidant potential (TRAP) was evaluated as an index of the non-enzymatic antioxidant capacity [16]. The results were expressed as the percentage of system area under curve inhibition (% AUC), as previously described [17]. Catalase (CAT) and superoxide dismutase (SOD) activities were analyzed in the cerebral cortex, hippocampus and striatum as a measure of the enzymatic antioxidant defenses [18,19]. These results were expressed as U CAT/mg protein and U SOD/mg protein.

### 2.4. Statistical Analyses

The data were analyzed using the Kruskal-Wallis test followed by Dunn’s post-hoc test. All data were analyzed using GraphPad Prism Software v.5.0 (GraphPad Software Inc., San Diego, CA, USA). The results were expressed as the mean ± standard deviation (S.D.); *p* values were considered significant when *p* ≤ 0.05.

## 3. Results

**Figure 1:**
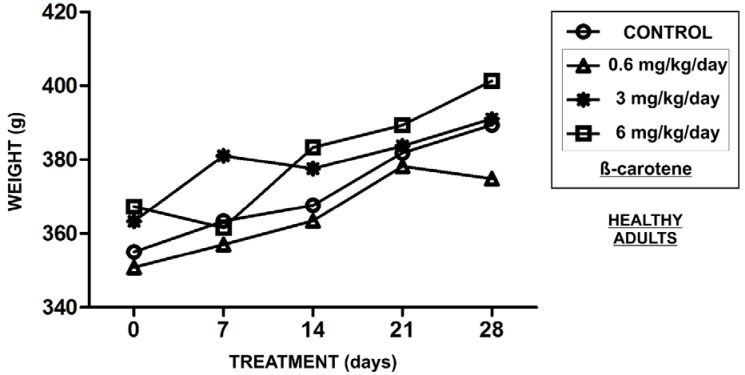
Absence of evidence of toxicity in β-carotene-supplemented healthy rats. A decrease in body weight in the treated animals was used as an indicator of *in vivo* toxicity. The results were considered significant when *p* < 0.05.

**Figure 2:**
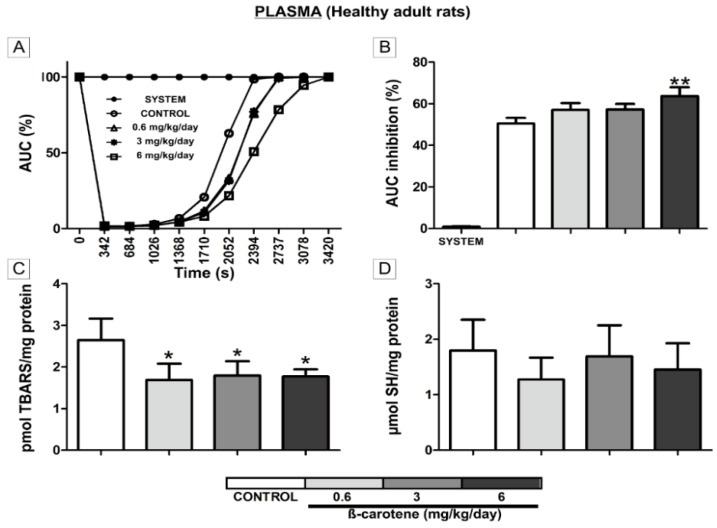
*In vivo* modulation of plasma redox parameters in β-carotene-supplemented adult healthy rats. Total reactive antioxidant potential (TRAP) in plasma from β-carotene-supplemented healthy animals when compared to controls (**A**,**B**). Lipid and protein damage parameters (TBARS and SH, respectively) detected in plasma from β-carotene-supplemented healthy animals when compared to controls (**C**,**D**). The results were considered significant when *p* < 0.05. * *p* ≤ 0.05; ** *p* ≤ 0.01.

The animals supplemented with β-carotene showed no treatment-related clinical signs of toxicity and no differences in body weight gain (Figure 1) during the experimentation. The β-carotene-supplemented animals also showed no gross lesions or abnormalities on necropsy examination. However, the plasma collected from the β-carotene-supplemented groups showed an increase in antioxidant activity, as demonstrated by the decrease in the AUC of the TRAP assay when compared to the control group (Figure 2A), but only the effect of the supplementation at 6 mg/kg/day was statistically significantly (*p* < 0.01) (Figure 2B). β-carotene supplementation at 0.6 (*p* < 0.05), 3 (*p* < 0.05), and 6 (*p* < 0.05) mg/kg/day also reduced the lipid peroxidation levels (TBARS) in the plasma, but did not affect the total thiol content (Figure 2C,D, respectively). We did not find any evidence for pro-oxidant activity in the brain induced by chronic β-carotene supplementation at these same doses (Table 1). In fact, no changes in CAT and SOD activities, TBARS levels, or SH content in the hippocampus and striatum were observed. However, supplementation at 3 mg/kg/day increased CAT activity in the cerebral cortex (*p* < 0.05) (Table 1).

**Table 1:** Oxidative stress parameters from selected brain regions of both control and β-carotene supplemented healthy rats. No pro-oxidant events were observed in the studied brain areas.

Redox Parameters	β-CAROTENE (mg/kg/day)			
	0 (Control)	0.6	3	6
**Number of supplemented rats**	6	7	7	7
**Hippocampus**				
TBARS level (nmol MDA/mg protein)	3.67 ± 0.66	3.29 ± 0.34	3.24 ± 0.53	2.96 ± 0.31
Total thiol content (mmol SH/mg protein)	17.7 ± 1.4	18.1 ± 0.8	17.8 ± 1.1	17.2 ± 1.3
CAT activity (U CAT/mg protein)	1.19 ± 0.21	1.04 ± 0.18	1.28 ± 0.22	1.09 ± 0.25
SOD activity (U SOS/mg protein)	32.1 ± 1.6	31.9 ± 0.8	33.4 ± 0.8	32.6 ± 1.7
**Striatum**				
TBARS level (nmol MDA/mg protein)	3.09 ± 0.73	2.63 ± 0.23	2.71 ± 0.56	2.67 ± 0.69
Total thiol content (mmol SH/mg protein)	17.7 ± 1.3	18.9 ± 1.9	20.4 ± 3.0	19.9 ± 2.5
CAT activity (U CAT/mg protein)	1.47 ± 0.6	1.16 ± 0.51	1.22 ± 0.48	0.96 ± 0.39
SOD activity (U SOD/mg protein)	32.7 ± 4.8	33.6 ± 2.9	36.1 ±2.2	33.6 ± 2.8
**Cerebral Cortex**				
TBARS level (nmol MDA/mg protein)	1.08 ± 0.18	1.3 ± 0.36	1.17 ± 0.35	0.99 ± 0.37
Total thiol content (mmol SH/mg protein)	22.4 ± 3.4	24.9 ± 3.1	21.9 ± 4.1	18.9 ± 4.9
CAT activity (U CAT/mg protein)	1.53 ± 0.37	1.86 ± 0.54	2.41 ± 0.39 *	1.79 ± 0.29
SOD activity (U SOD/mg protein)	37.9 ± 2.7	34.5 ± 4.4	37.4 ± 3.1	33.5 ± 5.1

* Significantly different from the control, *p* ≤ 0.05.

## 4. Discussion

Some clinical trials have already suggested a relationship between β-carotene consumption and higher incidences of lung cancer, together with an increase in the overall mortality when compared to placebo subjects [20,21]; however, several others did not find any adverse effects [22,23,24]. Doses equivalent to 30 mg of β-carotene have been associated with lung squamous metaplasia. However, doses on the order of 6 mg were able to provide moderate protection against the squamous metaplasia induced by cigarette smoke [25]. All these studies together may indicate that lower doses of β-carotene may potentially exert safe and beneficial effects on human health. In the present study, we utilized 0.6, 3, and 6 mg of β-carotene. These doses can be achieved not only with a regular diet rich in fruits and vegetables but also with dietary supplements, probably best reflecting the effects observed in healthy humans who are chronically exposed to β-carotene supplementation. In many Western countries, the current recommendation for vitamin A intake in adults is 600–1000 RAE/day. β-carotene supplementation may exceed these recommendations by factor of 4 [11,26,27]. Similarly, the nutritional requirement for Wistar rats is 700 RAE/kg/day, but the supplemented doses of 300, 600, and 3000 RAE/kg/day increased this value up to 4 times [28].

In this study, we observed that β-carotene supplementation, at the tested moderate doses, was able modify the plasma redox parameters in well-fed adult Wistar rats. These results contrast with other reports that expressed skepticism regarding the potential *in vivo* antioxidant properties of this supplement [29,30,31]. In fact, β-carotene increased TRAP and decreased TBARS levels without changing SH content in plasma after 28 days of supplementation. Because the TRAP results in plasma depend on the relative concentrations of antioxidants and their synergism, the observed modulations may indicate a successful absorption of β-carotene and improved antioxidant defense status [32,33]. Additionally, β-carotene is a lipophilic molecule, which tends to accumulate in lipophilic compartments such as membranes or lipoproteins, and has been postulated to be an important chain-breaking antioxidant, scavenging lipid oxide and lipid peroxide radicals [8]. It is also postulated that β-carotene is able protect human lymphocytes from damage caused by singlet oxygen, thereby protecting humans from the risk of several disorders, including cancer, cardiovascular, and ophthalmological diseases [34].

One could also speculate whether the numerous reported positive effects of β-carotene actions could be related, at least in part, to its redox modulatory characteristics in plasma as shown here in supplemented adult animals. It has been reported that β-carotene plays an important role in immune function and enhances lymphocyte proliferation independently of its provitamin A function [35]. Epidemiological studies also found a positive association between β-carotene intake and lung function, including higher forced expiratory volume and forced vital capacity [36]. Moreover, several epidemiological studies point towards an inverse association between β-carotene intake and cancer risk, especially at early stages of carcinogenesis [37]. The protective potential of β-carotene and other dietary antioxidants towards cancer and other oxidative stress-related diseases have been summarized in two important recent studies [38,39]. In contrast to the previously presented reports of the CARET [21] and ATBC [20] trials, there is a substantial body of data that generally supports the hypothesis that supplementation with β-carotene in non-smoker adults may reduce the morbidity associated with lung cancer [38]. Several human intervention trials also indicate that β-carotene is an important factor in preventing oral, pharyngeal, laryngeal, and esophageal cancer [38]. Similarly, case-control studies have confirmed an inverse relationship between the dietary intake of β-carotene and colon cancer [38]. In addition, the consumption of β-carotene seems to exert protective effects on cardiovascular diseases in 7 of 10 cohort studies, although no beneficial effect has been documented in any large-scale randomized trials such as ATBC, PHS, and WHS trials [39].

Overall, the results shown by a recent systematic review that examined all primary and secondary prevention randomized clinical trials involving β-carotene contradict the findings from the observational studies that claim that β-carotene improves health and protects against various diseases [40]. The explanation for the contradictions may be that the doses investigated in the clinical studies were higher than those that can be obtained from a diet rich in fruits and vegetables and even above the tolerable upper intake level [40]. Recently, it has been suggested that high doses of β-carotene may stimulate its asymmetric cleavage by non-enzymatic and enzymatic mechanisms including β-carotene-9′,10′-oxygenase (CMOII) [41]. It was also shown that CMOII behaves as a regulated oxidative stress protein, one of whose functions is to protect against the mitochondrial apoptosis induced by an excessive quantity of β-carotene [41]. However, stimulation of the asymmetric cleavage of β-carotene has been proposed to be responsible for the detrimental effects observed in many clinical trials because it may increase the levels of β-apocarotenoids [41]. The β-apocarotenoids may decrease the levels of retinoic acid and may modulate the signaling by nuclear receptors, which can lead to diminished retinoid signaling [41].

Our results encourage further exploration regarding the exact mechanisms by which redox modulation in plasma by β-carotene supplementation and the absence of pro-oxidant effects on the CNS are achieved. The present data suggest β-carotene as a safer (non-toxic) alternative option for nutritherapeutic vitamin A supplementation, usually focused on retinol esters that are associated with pro-oxidant-based neurotoxic effects on the CNS [4,5,6]. While our results show that no changes in SOD and CAT activities are induced by β-carotene supplementation, supplementation with retinol esters at 300, 600, and 3000 RAE/kg/day induces an imbalance in the SOD/CAT ratio in striatum, cerebral cortex, and hippocampus. This imbalance is also associated with increased lipoperoxidation (higher levels of TBARS) and a decreased SH content in the same CNS regions [4,5,6]. The nutritional alternative we present here (β-carotene supplementation) could have a critical impact on nutritherapeutic safety because oxidative stress in the CNS is associated with the development of several neuropsychiatric and neurodegenerative disorders including anxiety, depression, and Alzheimer’s disease [42].

Others have reported that supplementation with β-carotene attenuates the incidence of aneurysms and cerebral ischemia [43] and overcomes the association between a low intake of β-carotene and cognitive decline or an increased risk of dementia [44,45,46,47]. Longitudinal studies have also reported a lower rate of cognitive decline and a diminished risk of Alzheimer’s disease in subjects supplemented with β-carotene [48,49,50]. Furthermore, the Physician’s Health Study II (PHSII) reported that cognitive function benefits can be observed after long-term supplementation with β-carotene but following short-term intakes [51].

## 5. Conclusions

The improved antioxidant defense status of well-fed Wistar rats supplementation with β-carotene in the absence of any associated neurotoxic events suggest that β-carotene is a safer alternative source of vitamin A for food fortification and supplementation than the commonly used retinol esters. However, further work is needed to confirm our findings, to assess which metabolites are responsible for this modulation and determine the real potential of β-carotene as a nutritherapeutic supplement.
